# Dietary patterns and odds of prevalent gout comorbidity in diabetes mellitus: a mediation analysis of glucose metabolism and renal function

**DOI:** 10.7189/jogh.16.04280

**Published:** 2026-10-30

**Authors:** Xuelin He, Jiayan Zheng, Leyan Zheng, Guanghui Ying, Min Xia, Zhaogui Chen, Qiao Zhang, Baode Chen, Xia Liu, Yongtao Li

**Affiliations:** 1 Kidney Disease Center, the First Affiliated Hospital, Zhejiang University School of Medicine, Hangzhou, China; 2 Department of Nephrology, Beilun People's Hospital, Ningbo, China; 3 State Key Laboratory for Diagnosis and Treatment of Infectious Diseases, the First Affiliated Hospital, College of Medicine, Zhejiang University, Hangzhou, China; 4 Phase I Clinical Research Center, The First Affiliated Hospital of Zhejiang Chinese Medical University (Zhejiang Provincial Hospital of Traditional Chinese Medicine), Hangzhou, China; 5 Department of Laboratory Medicine, Key Laboratory of Clinical In Vitro Diagnostic Techniques of Zhejiang Province, The First Affiliated Hospital, College of Medicine, Zhejiang University, Hangzhou, China; 6 Institute for Intelligent Bio/ChemManufacturing (iBCM), Zhejiang University (ZJU)-Hangzhou Global Scientific and Technological Innovation Center, Hangzhou, China

**Keywords:** dietary patterns, diabetes mellitus, gout, glucose metabolism, renal function, Healthy Eating Index-2020, Dietary Approaches to Stop Hypertension, alternate Mediterranean diet

## Abstract

**Background:**

While independent associations between dietary patterns and diabetes mellitus (DM) or gout have been established, their relationship in comorbid conditions remains unclear. We aimed to examine the associations between distinct dietary patterns and prevalent gout among individuals with DM.

**Methods:**

We retrieved data in this cross-sectional study from the National Health and Nutrition Examination Survey. We used logistic regression models and Restricted cubic splines to explore associations between five dietary patterns (healthy eating index 2020 (HEI-2020), dietary approaches to stop hypertension (DASH), alternate Mediterranean diet score (aMED), dietary inflammatory index, and alternative healthy diet index). We also conducted subgroup and mediation analyses.

**Results:**

In multivariable-adjusted models, HEI-2020 (odds ratio (OR) = 0.82; 95% confidence interval (CI) = 0.70–0.96, *P* = 0.015), DASH (OR = 0.83; 95% CI = 0.70–0.98, *P* = 0.031), and aMED (OR = 0.86; 95% CI = 0.74–0.99, *P* = 0.041) scores were significantly associated with lower odds of prevalent gout among individuals with DM, with no nonlinear trends observed. Subgroup analyses revealed no significant interactions. Mediation analyses suggested that the triglyceride-glucose index-ratio of waist circumference to height mediated 40.2% and 22.8% of the effects of the DASH and HEI-2020 patterns, respectively. Serum uric acid showed evidence of mediation for the aMED pattern accounting for 16.3% of the association.

**Conclusions:**

Among people with DM in the USA, adherence to HEI-2020, DASH, and aMED dietary patterns is associated with lower odds of prevalent gout comorbidity in people with DM. Dietary structure improvement may therefore be associated with a lower burden of gout comorbidity in this population.

The escalating global prevalence of diabetes mellitus (DM), a metabolic disorder defined by chronic hyperglycaemia, constitutes a significant public health challenge [1]. Globally, an estimated 529 million individuals were living with DM in 2021 (age-standardised prevalence of 6.1%), a figure projected to surpass 1.31 billion by 2050 [2]. Individuals with DM exhibit comorbid metabolic syndromes, particularly gout [3]. Genetic correlation analyses reveal shared genetic factors between gout and type two DM, contributing to interdependent increases in morbidity [4], exacerbating disease complexity. Consequently, urgent measures are needed to mitigate gout risk in people with DM.

Dietary intervention is a critical, modifiable lever for preventing and managing both DM and gout [5,6]. Recent nutritional research has moved beyond isolated nutrients to dietary patterns that capture overall eating behaviour and its cumulative health impact [7]. Abundant evidence indicates that in specific populations, such as American adults, the Dietary Approaches to Stop Hypertension (DASH) and Alternate Mediterranean Diet Score (aMED) are inversely linked to the prevalence of both DM and gout [8,9]. Conversely, the Dietary Inflammatory Index (DII) score shows positive associations with the risk of both conditions [10,11]. However, existing studies have largely examined the independent associations of dietary patterns with either DM or gout, leaving the relationship between dietary patterns and their co-occurrence poorly understood. Notably, glucose metabolism and renal function are core pathophysiological mechanisms in DM and gout, respectively [12,13]. Whether dietary patterns are associated with comorbid diabetes-gout through these two key mechanisms remains unclear. Previous NHANES analyses have reported associations of the Healthy Eating Index-2015 (PMID: 35990332) and the Dietary Inflammatory Index (PMID: 38058438) with gout in the general US population, and a recent study further evaluated the association between the ketogenic diet ratio and gout among US adults with diabetes (PMID: 41556160). However, these studies focused on individual dietary indices and did not simultaneously compare multiple established dietary-quality indices in individuals with DM or explore potential metabolic, inflammatory, urate-related, and renal mediating pathways.

Therefore, using data from the National Health and Nutrition Examination Survey (NHANES) database, we investigated the association between different dietary patterns and prevalent gout among American adults with DM, and further explored the potential mediating roles of markers related to glucose metabolism and renal function, with the aim of providing evidence to inform dietary management in individuals with DM.

## METHODS

### Data sources

We derived data from the NHANES database, a nationwide survey by the National Centre for Health Statistics assessing the health and nutritional status of the United States population. The National Centre for Health Statistics Research Ethics Review Board approved the ethical protocol, and all participants provided informed consent. This study is a secondary analysis of NHANES data without direct human experimentation; therefore, it was exempt from additional institutional review board review. In accordance with JoGH’s GRABDROP reporting requirements, the required declarations regarding previous big-data publications, study originality, related analyses using the same data set, multiple testing, and the use of artificial intelligence are provided in Table S1 in the **Online Supplementary Document**.

We used aggregated data from six NHANES cycles (2007–2018; n = 34,770). After excluding participants without DM (n = 28,264) and those with incomplete data (n = 1439), 5,067 participants remained for the final analysis (**Figure 1**).

**Figure 1:**
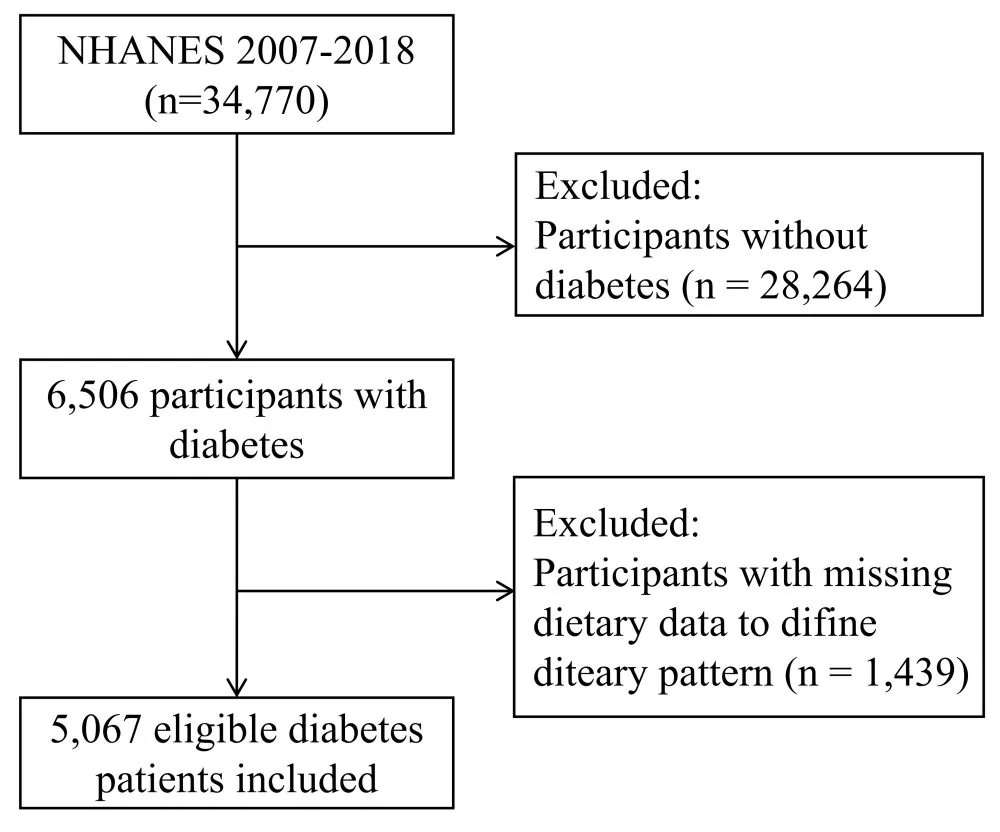
Participant selection flowchart.

### Dietary assessment

We derived dietary data from all participants through 24-hour dietary recall interviews. We included five dietary patterns and provided calculation methods for each (Methods S1 in the **Online Supplementary Document**).

The DASH score was computed from eight components: fruits, vegetables, nuts/legumes, low-fat dairy, whole grains, red/processed meats, sugar-sweetened beverages, and sodium [14]. The DII for the dietary inflammatory potential was obtained through 45 nutrients and food components; 39 were included in this study [15].

The Healthy Eating Index-2020 (HEI-2020) was assessed *via* 13 indicators: total vegetables, greens/beans, total fruits, whole fruits, dairy, whole grains, refined grains, total protein foods, seafood/plant proteins, fatty acid ratio, added sugars, saturated fats, and sodium [16].

The aMED was comprised of nine components: vegetables, fruits, whole grains, legumes, nuts, fish, the monounsaturated-to-saturated fat ratio, red/processed meats, and alcohol [17].

The Alternative Healthy Eating Index score was evaluated from 11 components: fruits, vegetables, whole grains, nuts/legumes, trans fat, red/processed meat, sugar-sweetened beverages, polyunsaturated fatty acids, docosahexaenoic acid and eicosapentaenoic acid, alcohol, and sodium [18].

### Disease definitions

DM was diagnosed from the American Diabetes Association criteria, defined as meeting any of the following: glycated haemoglobin ≥6.5%; 2-hour plasma glucose ≥200 mg/dL (75-g oral glucose tolerance test); fasting plasma glucose ≥126 mg/dL; a self-reported physician diagnosis; or self-reported consumption of insulin or other anti-diabetic agents [19]. Gout was determined by self-report in response to the question ‘Have you been diagnosed with gout by a doctor or other professionals?’ Participants answering ‘yes’ were defined as having gout [20].

### Mediating variables

Mediating variables included body mass index (BMI), estimated glomerular filtration rate (eGFR), serum uric acid (SUA), neutrophil-to-lymphocyte ratio (NLR), monocyte-to-lymphocyte ratio (MLR), triglyceride-glucose index (TyG), homeostatic model assessment for insulin resistance (HOMA-IR), triglyceride-glucose–body mass index (TyG-BMI), triglyceride-glucose–waist circumference (TyG-WC), and triglyceride-glucose–waist-to-height ratio (TyG-WHtR).

Covariates included sociodemographic characteristics, lifestyle habits, laboratory indices, and comorbidities (Table S2 in the **Online Supplementary Document**).

### Statistical analysis

All statistical analyses were performed using *R*, version 4.5.0 (R Foundation for Statistical Computing, Vienna, Austria). Dietary pattern scores were computed using the R package ‘dietaryindex’ (version 0.1.0) [21] using the first-day dietary recall weight (WTDRD1), divided by six to account for the pooling of six 2-year NHANES cycles. Subsequently, baseline characteristics were summarised using the R package ‘gtsummary’, version 2.1.0, with categorical variables as n (%) and continuous variables as mean (standard deviation (SD)). Weighted logistic regression models incorporating the complex survey design were fitted using the R package ‘survey’, version 4.4.2, to examine the associations between dietary patterns and prevalent gout among individuals with DM. Restricted Cubic Spline (RCS) analyses was performed using the R package ‘rms’, version 6.9.0, to examine potential nonlinear associations.

We used stratified multivariable logistic regression to quantify how dietary patterns affect the comorbidity of DM and gout and to assess the consistency of this association across clinically relevant subgroups. Finally, we conducted a complex survey-weighted mediation analysis using the product-of-coefficients method to explore potential pathways linking dietary patterns (HEI-2020, DASH, and aMED) to the odds of prevalent gout comorbidity in DM. Weighted regression models were constructed as follows: the a-path used weighted linear regression to assess the association between each dietary pattern and continuous mediators (including BMI, eGFR, SUA, NLR, and TyG-related indices); the b-path used weighted quasi-binomial logistic regression, adjusting for the same covariates plus the mediator, to evaluate the association between the mediator and the odds of prevalent gout. The indirect effect was estimated as the product of the a-path and b-path coefficients. The proportion mediated was calculated on the log-odds scale (indirect effect divided by total effect).

For mediators with missing data (*e.g.* some insulin metabolism indicators with high missingness), complete-case analysis was performed by constructing a weighted analytic sample for each mediator separately (Table S3 in the **Online Supplementary Document**). All models accounted for NHANES’s complex sampling design, including the stratification variable (SDMVSTRA) and primary sampling unit (SDMVPSU). We chose weights based on the analytic purpose: the main analyses (including RCS for dietary exposure and mediation analyses) used the two-day dietary weight (WTDR2D), whereas analyses involving fasting-related mediators (*e.g.* TyG indices) used the fasting subsample weight (WTSAF2YR). We estimated standard errors and 95% confidence intervals (CI) using Taylor series linearisation. To assess the potential impact of missing data, we also applied multiple imputation to key covariates and mediators (Table S4 in the **Online Supplementary Document**).

Analyses were conducted by implementing *R*, version 4.5.0 (R Foundation for Statistical Computing, Vienna, Austria); two-sided *P* < 0.05 was considered statistically significant for the primary association analyses. Because the subgroup and mediation analyses involved multiple comparisons, these analyses were considered exploratory; no formal multiplicity correction was applied, and nominal *P*-values were interpreted cautiously. Subgroup-specific associations were interpreted as potential effect modification only when supported by a statistically significant interaction test.

## RESULTS

### Baseline characteristics

The cohort comprised 5,067 participants with DM (weighted mean age: 59.65 years (SD = 13.69)), of whom 561 (10.5%) also had gout. Relative to those without gout, participants with gout demonstrated higher TyG-BMI, TyG-WC, TyG-WHtR, NLR, and MLR indices (*P* < 0.05), alongside a higher prevalence of male, aged ≥60 years, obesity, marriage, and comorbidities such as hypertension, cardiovascular disease (CVD), or chronic kidney disease (CKD) (*P* < 0.05) presented in **Table 1**.

**Table 1:** Characteristics of NHANES participants between 2007 and 2018

Characteristics	Overall (n = 5,067)	Non-gout (n = 4506)	Gout (n = 561)	*P*-value
Age, mean (SD)	59.65 (13.69)	59.00 (13.83)	65.10 (10.97)	<0.001
Older (≥60 years), n (%)	3,142.0 (53.0)	2,706.0 (50.9)	436.0 (70.7)	<0.001
Gender (male), n (%)	2,554.0 (49.7)	2,177.0 (47.9)	377.0 (64.8)	<0.001
Race, n (%)				<0.001
*Mexican American*	859.0 (9.7)	812.0 (10.3)	47.0 (4.2)	
*Non-Hispanic Black*	1,295.0 (14.2)	1,120.0 (14.1)	175.0 (15.8)	
*Non-Hispanic White*	1,915.0 (62.3)	1,659.0 (61.3)	256.0 (70.6)	
*Other Hispanic*	541.0 (5.9)	501.0 (6.1)	40.0 (4.0)	
*Other/multiracial*	457.0 (7.9)	414.0 (8.1)	43.0 (5.5)	
Education duration (>12 years), n (%)	3,453.0 (77.7)	3,041.0 (77.0)	412.0 (83.7)	0.002
Marital status (married), n (%)	2,782.0 (58.5)	2,445.0 (57.8)	337.0 (64.4)	0.038
PIR, n (%)				0.501
*<1.3*	1,590.0 (25.1)	1,417.0 (25.2)	173.0 (24.8)	
*1.3–3.5*	1,136.0 (36.0)	1,002.0 (35.6)	134.0 (39.5)	
*≥3.5*	1,885.0 (38.9)	1,672.0 (39.2)	213.0 (35.7)	
Obesity, n (%)	2,947.0 (64.4)	2,586.0 (63.4)	361.0 (72.7)	0.006
Current drink, n (%)	2,591.0 (53.2)	2,282.0 (52.8)	309.0 (56.0)	0.325
Current smoke, n (%)	801.0 (15.4)	738.0 (16.2)	63.0 (8.2)	<0.001
Physical activity (>600 METs/week), n (%)	2,188.0 (47.6)	1,986.0 (48.9)	202.0 (36.9)	0.002
Hypertension, n (%)	3,385.0 (65.8)	2,917.0 (63.7)	468.0 (83.1)	<0.001
Dyslipidaemia, n (%)	4,151.0 (83.1)	3,678.0 (82.7)	473.0 (86.7)	0.120
CVD, n (%)	1,294.0 (24.7)	1,048.0 (23.0)	246.0 (39.5)	<0.001
CKD, n (%)	890.0 (15.6)	697.0 (13.7)	193.0 (32.6)	<0.001
Insulin, mean (SD)	21.43 (27.94)	21.35 (28.40)	22.09 (23.54)	0.196
TyG, mean (SD)	9.15 (0.72)	9.14 (0.72)	9.29 (0.75)	0.150
HOMA-IR, mean (SD)	8.39 (13.22)	8.24 (13.09)	9.66 (14.27)	0.191
TyG-BMI, mean (SD)	300.27 (74.15)	297.26 (71.55)	326.11 (89.58)	0.016
TyG-WC, mean (SD)	1,012.23 (180.75)	1,003.41 (175.34)	1,091.06 (207.64)	0.001
TyG-WHtR, mean (SD)	6.06 (1.06)	6.02 (1.04)	6.41 (1.19)	0.017
HEI-2020, mean (SD)	51.88 (11.55)	51.93 (11.54)	51.42 (11.63)	0.537
aMED, mean (SD)	3.41 (1.27)	3.42 (1.28)	3.35 (1.24)	0.416
DASH, mean (SD)	22.57 (4.77)	22.59 (4.77)	22.36 (4.81)	0.521
AHEI, mean (SD)	39.17 (10.70)	39.15 (10.72)	39.35 (10.57)	0.928
DII, mean (SD)	2.90 (2.08)	2.91 (2.08)	2.80 (2.07)	0.276
NLR, mean (SD)	2.47 (1.40)	2.43 (1.34)	2.85 (1.81)	<0.001
MLR, mean (SD)	0.30 (0.14)	0.30 (0.14)	0.34 (0.16)	0.001
CRP, mean (SD)	6.36 (10.30)	6.23 (9.98)	7.56 (12.87)	0.702

AHEI – Alternative Healthy Eating Index, aMED – alternate Mediterranean diet, CKD – chronic kidney disease, CRP – C-reactive protein, CVD – cardiovascular disease, DASH – Dietary Approaches to Stop Hypertension, DII – Dietary Inflammatory Index, HEI-2020 – Healthy Eating Index-2020, HOMA-IR – homeostatic model assessment for insulin resistance, MLR – monocyte-to-lymphocyte ratio, NLR – neutrophil-to-lymphocyte ratio, PIR – Poverty Income Ratio, TyG – triglyceride-glucose index, TyG-BMI – triglyceride-glucose–body mass index, TyG-WC – triglyceride-glucose–waist circumference, TyG-WHtR – triglyceride-glucose-waist-to-height ratio.

### Association between dietary patterns and the odds of prevalent gout comorbidity in DM

For multivariable analysis, in the fully adjusted model, HEI-2020 (OR = 0.82; 95% CI = 0.70–0.96, *P* = 0.015), DASH (OR = 0.83; 95% CI = 0.70–0.98, *P* = 0.031), and aMED (OR = 0.86; 95% CI = 0.74–0.99, *P* = 0.041) scores were associated with lower odds of prevalent gout comorbidity in DM (**Table 2**). Following multivariable adjustment, RCS results indicated significant overall trends for HEI-2020 (*P*-overall = 0.005), DASH (*P*-overall = 0.007), and aMED (*P*-overall = 0.035), but no nonlinear associations were detected (*P*-nonlinearity >0.05) (**Figure 2**). Sensitivity analyses using multiple imputation showed that the direction and magnitude of the total effects of HEI-2020, DASH, and aMED on the odds of prevalent gout were consistent with those from complete-case analyses (Figure S1, Table S5 in the **Online Supplementary Document**).

**Table 2:** The odds ratios between dietary pattern and gout in diabetes patients, NHANES 2007–2018

Dietary patterns	HEI-2020			AHEI		DASH		aMED		DII	
		**OR (95% CI)**	***P*-value**	**OR (95% CI)**	***P*-value**	**OR (95% CI)**	***P*-value**	**OR (95% CI)**	***P*-value**	**OR (95% CI)**	***P*-value**
Unweighted SD (score points)		11.73		10.56		4.63		1.28		2.10	
Crude model		0.96 (0.83–1.10)	0.511	1.02 (0.89–1.16)	0.777	0.95 (0.82–1.11)	0.526	0.94 (0.83–1.07)	0.366	0.95 (0.82–1.10)	0.453
Model 1*		0.85 (0.74–0.97)	0.017	0.90 (0.78–1.03)	0.121	0.84 (0.71–0.98)	0.026	0.85 (0.74–0.97)	0.019	1.06 (0.89–1.26)	0.508
Model 2†		0.84 (0.73–0.96)	0.013	0.90 (0.78–1.03)	0.128	0.83 (0.70–0.97)	0.024	0.85 (0.74–0.97)	0.017	1.06 (0.90–1.26)	0.474
Model 3‡		0.82 (0.70–0.96)	0.015	0.95 (0.78–1.15)	0.594	0.83 (0.70–0.98)	0.031	0.86 (0.74–0.99)	0.041	1.01 (0.80–1.26)	0.943

AHEI – Alternative Healthy Eating Index, aMED – alternate Mediterranean diet, CI – confidence interval, CVD – cardiovascular disease, DASH – Dietary Approaches to Stop Hypertension, DII – Dietary Inflammatory Index, HEI-2020 – Healthy Eating Index-2020, OR – odds ratio, SGLT2 – sodium-glucose cotransporter 2, SD – standard deviation

ORs are presented per 1-SD increase in dietary scores, using unweighted SDs.

*Model 1: Adjusted for age (years), gender, race/ethnicity, education duration, and marital status.

†Model 2: Adjusted for Model 1 plus alcohol consumption, smoking status, leisure-time physical activity, and total energy intake (kcal/d).

‡Model 3: Adjusted for Model 2 plus hypertension, CVD, dyslipidemia, obesity, and medication use (including urate-lowering drugs, diuretics, SGLT2 inhibitors, and other anti-diabetic medications).

**Figure 2:**
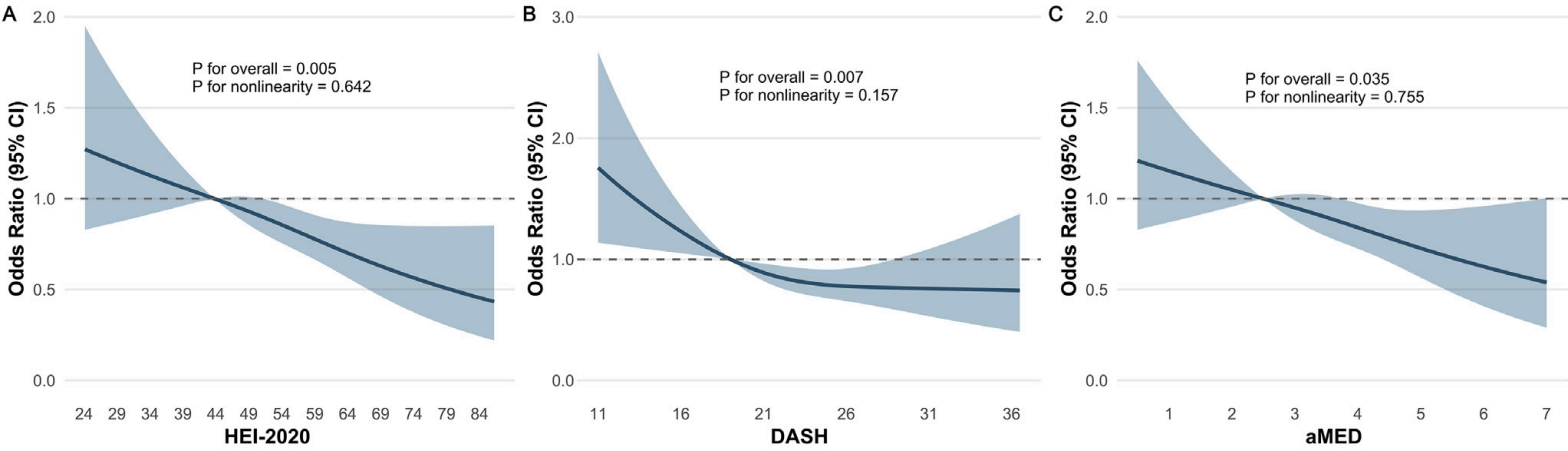
Dose-response relationship between dietary pattern scores and gout prevalence in people with diabetes, NHANES 2007–2018.

### Subgroup analysis

Subgroup analyses showed that the direction of associations between dietary patterns and the odds of prevalent gout among individuals with DM was generally consistent across population strata. In the CVD subgroup, higher HEI-2020 scores were significantly associated with lower odds of prevalent gout among individuals without CVD (OR = 0.65; 95% CI = 0.48–0.87, *P* = 0.005). Among people with chronic kidney disease (CKD), higher aMED scores were associated with lower odds of prevalent gout (OR = 0.81; 95%CI = 0.68–0.97, *P* = 0.025), whereas no similar association was observed in the non-CKD population. Additionally, in people who smoked, the negative association between aMED and the odds of prevalent gout was more significant (OR = 0.58; 95% CI = 0.40–0.86, *P* = 0.007) (**Table 3**).

**Table 3:** Subgroup analysis for the association between dietary pattern and gout in diabetes patients, NHANES 2007–2018

			HEI-2020			DASH			aMED	
**Subgroup**	**Gout/non-gout, n**	**OR (95%CI)**	***P*-value**	***P* for interaction**	**OR (95%CI)**	***P*-value**	***P* for interaction**	**OR (95%CI)**	***P*-value**	***P* for interaction**
Gender				0.979			0.409			0.671
*Male*	377/2,177	0.80 (0.64–1.00)	0.055		0.77 (0.58, 1.02)	0.070		0.81 (0.68, 0.97)	0.024	
*Female*	184/2,329	0.84 (0.67–1.05)	0.115		0.98 (0.71, 1.37)	0.921		0.91 (0.70, 1.19)	0.503	
Age group				0.805			0.325			0.609
*<60 years*	125/1,800	0.81 (0.68–0.98)	0.030		0.78 (0.62, 0.98)	0.032		0.81 (0.67, 0.98)	0.033	
*≥60 years*	436/2,706	0.87 (0.64–1.18)	0.363		1.03 (0.68, 1.56)	0.898		0.97 (0.72, 1.31)	0.846	
Current smoke				0.491			0.534			0.065
*No*	498/3,768	0.83 (0.70–0.98)	0.028		0.85 (0.68, 1.06)	0.148		0.88 (0.75, 1.03)	0.120	
*Yes*	63/738	0.74 (0.51–1.09)	0.122		0.84 (0.56, 1.26)	0.396		0.58 (0.40, 0.86)	0.007	
Current drink				0.522			0.491			0.478
*No*	252/2,224	0.89 (0.73, 1.10)	0.284		0.98 (0.75, 1.29)	0.889		0.96 (0.77, 1.19)	0.709	
*Yes*	309/2,282	0.77 (0.62, 0.96)	0.023		0.77 (0.59, 1.00)	0.051		0.79 (0.66, 0.94)	0.011	
Physical Activity				0.639			0.933			0.805
*<600 Mets/week*	359/2,520	0.78 (0.64, 0.96)	0.020		0.81 (0.63, 1.06)	0.123		0.83 (0.68, 1.01)	0.068	
*≥600 Mets/week*	202/1,986	0.88 (0.70, 1.11)	0.285		0.92 (0.62, 1.35)	0.659		0.90 (0.70, 1.17)	0.439	
Obesity				0.678			0.573			0.315
*Yes*	361/2,586	0.88 (0.68, 1.14)	0.331		0.95 (0.69, 1.31)	0.763		0.97 (0.79, 1.20)	0.794	
*No*	183/1,833	0.81 (0.67, 0.99)	0.037		0.82 (0.63, 1.06)	0.133		0.82 (0.68, 0.99)	0.034	
Hypertension				0.073			0.289			0.166
*Yes*	468/2,917	1.04 (0.78, 1.39)	0.788		0.98 (0.58, 1.67)	0.950		1.01 (0.68, 1.50)	0.959	
*No*	93/1,589	0.77 (0.63, 0.93)	0.007		0.80 (0.64, 1.00)	0.053		0.82 (0.70, 0.97)	0.018	
CVD				0.064			0.228			0.258
*Yes*	246/1,048	0.94 (0.75, 1.17)	0.564		0.93 (0.68, 1.25)	0.614		0.90 (0.73, 1.10)	0.290	
*No*	315/3,458	0.65 (0.48, 0.87)	0.005		0.75 (0.55, 1.01)	0.059		0.80 (0.61, 1.05)	0.105	
CKD				0.388			0.055			0.057
*Yes*	198/841	0.81 (0.64, 1.02)	0.075		0.78 (0.59, 1.05)	0.101		0.81 (0.68, 0.97)	0.025	
*No*	307/3,426	0.96 (0.71, 1.31)	0.806		1.27 (0.94, 1.70)	0.112		1.22 (0.89, 1.67)	0.212	

aMED – alternate Mediterranean diet, CI – confidence interval, CKD – chronic kidney disease, CVD – cardiovascular disease, DASH – Dietary Approaches to Stop Hypertension, HEI-2020 – Healthy Eating Index-2020, OR – odds ratio

Overall, although we observed differential associations in some subgroups, we detected no statistically significant interaction. Therefore, interpret these results cautiously, mainly as exploratory analyses that provide clues for potential effect modification.

### Mediation analysis

Mediation analyses revealed differentiated mediating roles of systemic inflammation, uric acid metabolism, and insulin resistance-related indices in the associations between healthy dietary patterns and lower odds of prevalent gout among individuals with DM (Table S6 in the **Online Supplementary Document**). In the immune-inflammatory pathway, NLR showed significant mediation for all three dietary patterns (DASH = 8.0%; HEI-2020 = 6.0%; aMED = 7.1%). In the metabolic and insulin resistance pathway, the composite index TyG-WHtR exhibited strong mediation, explaining 40.2% (95% CI = 2.1%–78.3%, *P* = 0.039) of the DASH effect and 22.8% (95% CI = 0.7%–44.9%, *P* = 0.044) of the HEI-2020 effect, whereas the single TyG index did not reach statistical significance. SUA showed significant mediation only for the aMED pattern, accounting for 16.3% (95% CI = 0.5%–32.0%, *P* = 0.043), and was not significant for HEI-2020. Multiple-imputation analyses yielded broadly consistent mediation patterns: the significant mediation effects of NLR across all three dietary patterns, TyG-WHtR for DASH and HEI-2020, and SUA for aMED were retained (Table S7 in the **Online Supplementary Document**).

## DISCUSSION

We found that higher HEI-2020, DASH, and aMED scores were significantly associated with lower odds of prevalent gout comorbidity among American adults with DM. Mediation analyses further suggested that TyG-WHtR, SUA, and NLR showed significant mediation for specific dietary patterns. We used the Chronic Kidney Disease Epidemiology Collaboration 2021 equation without a race coefficient to estimate eGFR, avoiding potential bias from race adjustment. This approach might produce slightly lower eGFR estimates for non-Hispanic Black participants than traditional equations, likely reflecting their kidney function more accurately.

HEI-2020, DASH, and aMED scores were inversely associated with the odds of prevalent gout comorbidity among individuals with DM. HEI-2020 serves as a diet-quality metric derived from the Dietary Guidelines for Americans, where higher scores reflect greater adherence to a healthy diet [16]. The DASH diet prioritises intake of vegetables, fruits, nuts, legumes, whole grains, and low-fat dairy, while restricting sugar-sweetened beverages, sodium, and red/processed meats [22]. Similarly, the aMED pattern advocates vegetable, fruit, and whole-grain consumption, incorporates the monounsaturated-to-saturated fat ratio, and limits red/processed meats [23]. Previous studies in USA populations have reported that higher DASH and aMED scores are associated with a lower risk of incident gout [9,24]. Although no study has directly linked HEI-2020 to gout, its dietary structure shares metabolic regulatory potential with DASH and aMED.

Consequently, we hypothesise that these dietary patterns may reduce the odds of gout comorbidity in DM through multiple mechanisms, including shared anti-inflammatory and antioxidant properties, improved insulin sensitivity, and restriction of high-purine foods. Gout is an inflammatory arthritis [25]. Under the influence of the HEI-2020, DASH, and aMED dietary patterns, the body develops a comprehensive anti-inflammatory and antioxidant environment [26–28] that may render joint tissues less susceptible to intense inflammatory responses triggered by urate crystals, reducing the incidence of gout episodes in DM [29]. These patterns have also been confirmed to improve insulin sensitivity for those managing DM [30–31], potentially benefiting uric acid excretion and reducing the incidence of gout [33]. Furthermore, these patterns limit the intake of high-purine foods, reducing the endogenous production of uric acid [34]. In summary, this study extends the evidence for the beneficial effects of diet on gout to individuals with DM, a high-risk group sharing common pathophysiological foundations, potentially providing important strategies for gout management and comprehensive metabolic regulation in this population. Notably, the multiple dietary indices employed in this study have overlapping components; the consistent associations likely reflect a common effect of overall dietary quality rather than independent effects of any specific dietary pattern. Future research should attempt to construct streamlined or de-overlapped dietary indices to further explore whether unique components of each dietary pattern possess independent discriminative value.

Additionally, higher HEI-2020 scores were associated with lower odds of prevalent gout comorbidity in DM among people without CVD. Conversely, higher aMED scores were associated with lower odds of prevalent gout only in individuals with CKD. These subgroup differences may reflect variations in comorbidity status and treatment across populations. Patients with CVD often have higher baseline inflammatory burden and commonly use anti-inflammatory medications such as statins and aspirin, which may mask or dilute the independent protective effects of dietary patterns [35,36]. In contrast, people without CVD have not yet developed severe cardiovascular organic lesions and exhibit relatively lower inflammation levels, thus better reflecting the low-inflammatory potential of HEI-2020. In addition, patients with CKD have marked uric acid excretion impairment and chronic inflammation; the aMED, rich in polyphenols and unsaturated fatty acids, may exert protective effects by improving kidney function and reducing oxidative stress [37–39], whereas such effects offer limited marginal benefit in non-CKD individuals with normal kidney function.

Similarly, among people who smoked, higher aMED scores were associated with lower odds of prevalent gout comorbidity in DM. Smoking is known to induce systemic oxidative stress and chronic low-grade inflammation, promote uric acid production, and impair kidney function [40,41], indirectly increasing the odds of prevalent gout. Foods rich in monounsaturated fatty acids and high-antioxidant vegetables and fruits in the aMED may partially alleviate smoking-induced oxidative damage through anti-inflammatory and antioxidant actions [42,43]. Therefore, among people with DM who continue to smoke or have recently quit, adherence to the aMED pattern may reduce gout comorbidity odds more effectively by lowering the inflammatory burden. However, the stability of these findings may be limited by the distribution of subgroup characteristics after stratification; thus, these analyses are primarily exploratory and aim to identify potential effect modifiers and generate hypotheses for future research.

Mediation analyses revealed that the associations between different healthy dietary patterns and lower odds of prevalent gout comorbidity in DM may operate through several biologically relevant pathways. Notably, the association between the DASH pattern and TyG-WHtR explained up to 40.2% of the effect on lower odds of prevalent gout comorbidity, and the HEI-2020 pattern contributed 22.8% *via* this pathway. These findings suggest that higher DASH and HEI-2020 scores may partially reduce gout odds by improving metabolic disturbances related to insulin resistance and central obesity [32,44–46]. However, TyG-WHtR is a composite indicator reflecting multiple physiological processes; interpret its mediation cautiously in terms of a single molecular mechanism. In contrast, the aMED pattern exhibited a specific association with SUA levels, with SUA mediating 16.3% of its effect on lower odds of prevalent gout comorbidity. This observation aligns with previous studies linking higher DASH and aMED scores to lower SUA levels [47,48], possibly through preserved renal function (higher eGFR) [49,^50^]. Furthermore, systemic inflammation showed modest but significant mediation (6.0–8.0%) across all three dietary patterns, suggesting that anti-inflammatory dietary characteristics may be associated with lower odds of prevalent gout comorbidity. Importantly, missing data in this study may influence the mediation effect estimates, particularly for metabolic variables with some missingness, which may reduce statistical power and affect the precision of the estimates. Therefore, these results should be regarded primarily as exploratory observations of potential biological pathways rather than as evidence for causal inference, and they should be interpreted with caution. Future studies with more complete data collection and sensitivity analyses are needed to further validate these findings.

However, several limitations exist. First, analyses were restricted to a cross-sectional sample of USA population. Heterogeneity across countries in dietary pattern composition, gout diagnostic criteria, and DM care pathways suggests the findings may not generalise directly to diabetic patients of other races, cultural backgrounds, or healthcare systems. Validation studies in diverse populations are needed. Second, gout in this study was based on self-reported physician diagnosis, which may introduce misclassification bias that could vary across population subgroups and potentially affect association estimates. Therefore, interpret the results cautiously. Additionally, the study did not distinguish between type one and type two diabetes; the results mainly reflect the overall population with DM. Although the models adjusted for medication use and other factors, residual confounding cannot be fully excluded, and results should be interpreted with caution. Furthermore, using weighted odds ratios to estimate associations between dietary patterns and gout may overestimate relative risk when outcome prevalence is high and does not reflect absolute risk or population-level disease burden. Third, due to limitations of the NHANES database, the study could not conduct sensitivity analyses focusing on participants using urate-lowering drugs or those with severely impaired kidney function. Although urate-lowering therapy was included as a covariate in the main models, the potential direct interference of other medications with SUA as a mediator cannot be fully excluded. Moreover, the cross-sectional design can only reveal associations between variables, cannot establish causal relationships, and cannot determine the temporal sequence of exposure and outcome, leaving the possibility of reverse causation. Fourth, no formal adjustment for multiple comparisons was applied to the subgroup or mediation analyses. These analyses should therefore be regarded as exploratory, and nominal *P* values, particularly those close to 0.05, should be interpreted cautiously. In addition, some subgroups had limited case numbers across multiple strata, potentially reducing statistical power. In addition, missing data were handled by multiple imputation and the imputed results were consistent with the main analyses. However, the potential influence of unmeasured factors on fasting subsample selection cannot be completely ruled out. Finally, although we examined putative mediators, we did not thoroughly investigate the underlying biological mechanisms. Future research should involve more in-depth experiments and analyses to uncover these mechanisms.

## CONCLUSIONS

Among USA adults with DM, higher HEI-2020, DASH, and aMED dietary pattern scores are associated with lower odds of prevalent gout comorbidity. These associations may be partially mediated through pathways related to insulin resistance and central adiposity, systemic inflammation, and serum uric acid metabolism, as reflected by TyG-WHtR, NLR, and SUA. Therefore, greater adherence to these healthy dietary patterns may be associated with lower odds of prevalent gout comorbidity among adults with DM.

## Additional material


Online Supplementary Document

## Data Availability

**Data availability:** The data sets supporting the findings of this study are publicly available through the National Health and Nutrition Examination Survey website (https://wwwn.cdc.gov/nchs/nhanes/default.aspx).
