# Supplementary material for: Dietary patterns and odds of prevalent gout comorbidity in diabetes mellitus: a mediation analysis of glucose metabolism and renal function
Source: J Glob Health. 2026 Oct 30;16:04280. doi: 10.7189/jogh.16.04280 (PMC13721645; doi:10.7189/jogh.16.04280)
Supplement: Supplementary file 1 [file jogh-16-04280-s001.pdf]

Supplement to: Xuelin Hea, Jiayan Zheng, Leyan Zheng, Guanghui Ying, Min Xia, Zhaogui Chen, Qiao Zhang, Baode Chen, Xia Liu, Yongtao Li. Dietary patterns and odds of prevalent gout comorbidity in diabetes mellitus: a mediation analysis of glucose metabolism and renal function. J Glob Health. 2026;16:04280.

Supplementary Methods

Dietary Approaches to Stop Hypertension (DASH) Diet: The DASH score was constructed based on the consumption of foods and nutrients emphasized or minimized in the DASH diet, focusing on eight components: fruits, vegetables, nuts/legumes, whole grains, low-fat dairy products, red/processed meats, sugar-sweetened beverages, and sodium. For each participant, energy-adjusted intake of the aforementioned foods and nutrients was calculated using the residual method[1]. Subsequently, participants were categorized into corresponding deciles according to these energy-adjusted intake values. For fruits, vegetables, nuts/legumes, whole grains, and low-fat dairy products, participants in the highest decile were assigned a score of 10, while those in the lowest decile were assigned a score of 1. Conversely, for red/processed meats, sugar-sweetened beverages, and sodium, individuals in the highest decile were assigned a score of 1, and those in the lowest decile received a score of 10. Finally, the DASH total score for each participant, ranging from 8 to 80, was derived by summing the scores of these eight components. A higher total score indicates a dietary pattern closer to the ideal DASH model[2].

Dietary Inflammatory Index (DII): The DII involves 45 food parameters in total, with each parameter assigned a specific DII score based on its effect on six key inflammatory biomarkers: IL-1β, IL-4, IL-6, IL-10, TNF-α, and CRP. In the present study, 39 out of the 45 food parameters were included: alcohol, vitamin B12, vitamin B6, β-carotene, caffeine, carbohydrates, cholesterol, energy, total fat, fiber, folate, iron, magnesium, monounsaturated fatty acids, niacin, n-3 fatty acids, n-6 fatty acids, protein, polyunsaturated fatty acids, vitamin B2, saturated fatty acids, selenium, vitamin B1, vitamin A, vitamin C, vitamin E, zinc, vitamin D, garlic, ginger, onion, flavan-3-ol, flavonoids, flavonols, flavanones, anthocyanidins, isoflavones, pepper, and thyme. The overall DII value, calculated by summing the scores of all specific food components, was used to assess the inflammatory potential of one participant’s diet, where positive values denote a pro-inflammatory effect, negative values an anti-inflammatory effect, and zero signifies neutrality[3].

Healthy Eating Index 2020 (HEI-2020): The HEI-2020 comprises 13 distinct components, including adequacy components (total vegetables, greens/beans, total fruits, whole fruits, whole grains, dairy, total protein foods, seafood/plant proteins, and fatty acid ratio) and moderation components (sodium, refined grains, saturated fats, and added sugars). Each component was assigned a unique weight and a different maximum score. The HEI-2020 score ranges from 0 to 100, with higher scores indicating better diet quality[4].

Alternate Mediterranean Diet Score (aMED): The aMED includes 9 dietary components: vegetables, fruits, whole grains, legumes, nuts, fish, the ratio of monounsaturated to saturated fats, red/processed meats, and alcohol. Each component was scored as 0, 0.5, or 1 point. For vegetables (excluding potatoes), fruits, whole grains, legumes, nuts, fish, and the monounsaturated-to-saturated fat ratio, scores were assigned as follows: 0 points for no intake, 0.5 points for intake below the median, and 1 point for intake above the median. For red/processed meat intake, a score of 0 was assigned for intake above the median, 0.5 points for intake at or below the median, and 1 point for no intake. For alcohol, a score of 1 was assigned for moderate intake, and 0 points for excessive intake or no alcohol consumption. The total score ranges from 0 to 9, with a higher total score indicating greater adherence to the aMED pattern[5].

Alternative Healthy Eating Index (AHEI): The AHEI consists of 11 components: 6 healthy components where higher intake is better (vegetables, fruits, whole grains, nuts and legumes, docosahexaenoic acid and eicosapentaenoic acid [DHA/EPA], polyunsaturated fatty acids), 1 component where moderate intake is optimal (alcohol), and 4 reverse-scored components that should be limited or avoided (sugar-sweetened beverages and fruit juice, red/processed meat, trans fat, sodium). Each component was scored on a scale from 0 to 10 points. For the six healthy indicators, higher intake received higher scores. For the four reverse-scored indicators, lower consumption obtained higher scores. For alcohol: women consuming ≥2.5 servings/day scored 0, while intake of 0.5–1.5 servings/day scored the maximum; men consuming ≥3.5 servings/day scored 0, while intake of 0.5–2.0 servings/day scored the maximum. The

total score ranges from 0 to 110, with a higher total score indicating a healthier dietary pattern[6].

Reference

1. Willett WC, Howe GR, Kushi LH. Adjustment for total energy intake in epidemiologic studies. Am J Clin Nutr. 1997; 65:1220S-8S; discussion 9S-31S.
2. Soltani S, Hashemi R, Heshmat R, Motlagh AD, Esmaillzadeh A. Association of dietary approaches to stop hypertension eating style and risk of sarcopenia. Sci Rep. 2020; 10:19339.
3. Shivappa N, Steck SE, Hurley TG, Hussey JR, Hebert JR. Designing and developing a literature-derived, population-based dietary inflammatory index. Public Health Nutr. 2014; 17:1689-96.
4. Shams-White MM, Pannucci TE, Lerman JL, Herrick KA, Zimmer M, Meyers Mathieu K, et al. Healthy Eating Index-2020: Review and Update Process to Reflect the Dietary Guidelines for Americans,2020-2025. J Acad Nutr Diet. 2023; 123:1280-8.
5. Fung TT, Rexrode KM, Mantzoros CS, Manson JE, Willett WC, Hu FB. Mediterranean diet and incidence of and mortality from coronary heart disease and stroke in women. Circulation. 2009; 119:1093-100.
6. Chiuve SE, Fung TT, Rimm EB, Hu FB, McCullough ML, Wang M, et al. Alternative dietary indices both strongly predict risk of chronic disease. J Nutr. 2012; 142:1009-18.

Table S1. Adherence to JoGH's Guidelines for Reporting Analyses of Big Data Repositories Open to the Public (GRABDROP)

GRABDROP item	Authors' statement
1. Previous publications based on secondary analyses of large public data repositories	None of the authors has published, within the past three years, a study based on secondary analysis of a large publicly accessible data repository.
2. Originality of the present study	Our study focused specifically on U.S. adults with diabetes mellitus and evaluated five established dietary indices—HEI-2020, DASH, aMED, AHEI, and DII—within the same survey-weighted NHANES framework. We further assessed potential nonlinear associations and explored whether markers related to glucose metabolism, systemic inflammation, uric acid metabolism, and renal function might mediate the observed associations. The simultaneous comparison of multiple dietary patterns together with mediation analyses in adults with diabetes represents the main original contribution of this study.
3. Related studies using the same data repository	Previous NHANES studies addressing related research questions include: (1) Nie et al., who examined the association between HEI-2015 and gout/hyperuricemia; (2) Zhang et al., who evaluated the association between the Dietary Inflammatory Index and gout; and (3) Zheng et al., who investigated the association between the ketogenic diet ratio and gout among U.S. adults with diabetes. These studies are cited and discussed in the Introduction [PMID: 35990332; PMID: 38058438; PMID: 41556160]. The present study differs by simultaneously evaluating multiple established dietary-quality indices in adults with diabetes and further exploring potential metabolic, inflammatory, urate-related, and renal mediating pathways.
4. Multiple testing	We used a two-sided P value <0.05 for the primary association analyses and did not apply a formal multiplicity correction. Because the subgroup and mediation analyses involved multiple comparisons, these analyses were considered exploratory and their nominal P values were interpreted cautiously. Subgroup-specific findings were interpreted as evidence of effect modification only when supported by a statistically significant interaction test.

GRABDROP item	Authors' statement
5. Use of artificial intelligence tools	Generative artificial intelligence tools were used during manuscript revision to assist with language editing, sentence restructuring, consistency checking, and proofreading. They were not used to generate, modify, or analyse the study data or statistical results. All AI-assisted text was reviewed and revised by the authors, who take full responsibility for the content of the final manuscript.

Table S2. Specific information and definitions of covariates

Covariates	
Age	
	<60 years
	⩾60 years
Gender	
	Male
	Female
Race	
	Mexican American
	Non-Hispanic Black
	Non-Hispanic White
	Other Hispanic
	Other/multiracial
Education duration	
	⩽12 years
	>12 years
Marital status	
	Unmarried
	Married
PIR	
	< 1.3
	1.3-3.5
	≥ 3.5
Obesity	BMI≥30 kg/m²
Drink	
	Non-drink
	Current drink (>1 drink/month)
Smoke	
	Non-smoke (<100 cigarettes/lifetime)
	Current smoke (≥100 cigarettes/lifetime)
Physical Activity	
	<600METs/week
	≥600METs/week
Total energy intake	
	Calculated as the average daily caloric intake (kcal/day) derived from two 24-hour dietary recall interviews.

Hypertension	Defined as meeting any of the following criteria: (1) Self-reported physician diagnosis of hypertension; (2) Current use of antihypertensive medication; or (3) Measured systolic blood pressure >140 mmHg or diastolic blood pressure >90 mmHg.
Dyslipidemia	Defined as meeting any of the following criteria: (1) Total cholesterol ≥240 mg/dL; (2) LDL-C ≥160 mg/dL; (3) HDL-C <40 mg/dL; (4) Triglycerides ≥200 mg/dL; or (5) Current use of lipid-lowering medication.
CVD	Defined as meeting any of the following criteria based on self-report: (1) Myocardial infarction; (2) Congestive heart failure; (3) Coronary heart disease; (4) Angina pectoris; or (5) Stroke.
CKD	Defined as meeting any of the following criteria: (1) UACR ≥30 mg/g; or (2) eGFR <60 mL/min/1.73 m².
urate-lowering drugs	Identified via the Prescription Drug Questionnaire. Defined as the self-reported use of xanthine oxidase inhibitors or uricosuric agents within 30 days prior to the interview.
diuretics	Identified via the Prescription Drug Questionnaire. Defined as the use of thiazide diuretics, loop diuretics, or potassium-sparing diuretics within 30 days prior to the interview.
SGLT2 inhibitors	Identified by generic drug names within the Prescription Drug Questionnaire. Defined as the use of sodium-glucose cotransporter 2 inhibitors within 30 days prior to the interview.
anti-diabetic medications	Identified through the Prescription Drug Questionnaire and the Diabetes Questionnaire. Defined as meeting either of the following criteria: (1) use of oral hypoglycemic agents or insulin within 30 days prior to the interview; (2) self-reported current use of medication to lower blood glucose.

Abbreviations: BMI – body mass index; CKD – chronic kidney disease; CVD – cardiovascular disease; eGFR – estimated glomerular filtration rate; HDL-C – high-density lipoprotein cholesterol; LDL-C – low-density lipoprotein cholesterol; PIR – Poverty Income Ratio; SGLT2 – sodium-glucose cotransporter 2; UACR – urinary albumin-to-creatinine ratio.

Table S3. Summary of Missing Data Distribution for Study Variables

Variable	Missing Count	Missing Percentage
TyG-WHtR	2511	49.56
TyG-WC	2504	49.42
TyG-BMI	2422	47.8
TyG	2370	46.77
HOMA-IR	2368	46.73
Insulin	2363	46.64
Glucose	2294	45.27

CRP	1623	32.03
PIR	456	9
CKD	296	5.84
LBXSUA	250	4.93
eGFR	249	4.91
NLR	159	3.14
MLR	159	3.14
BMI	104	2.05
Age	0	0
Gender	0	0
Race	0	0
Education duration	0	0
Marital status	0	0
Current drink	0	0
Current smoke	0	0
Physical Activity	0	0
Hypertension	0	0
Dyslipidemia	0	0
CVD	0	0
HEI-2020	0	0
aMED	0	0
DASH	0	0
AHEI	0	0
DII	0	0

Abbreviations: AHEI – Alternative Healthy Eating Index; aMED – alternate Mediterranean diet; BMI – body mass index; CKD – chronic kidney disease; CRP – C-reactive protein; CVD – cardiovascular disease; DASH – Dietary Approaches to Stop Hypertension; DII – Dietary Inflammatory Index; eGFR – estimated glomerular filtration rate; HEI-2020 – Healthy Eating Index-2020; HOMA-IR – homeostatic model assessment for insulin resistance; LBXSUA – serum uric acid; MLR – monocyte-to-lymphocyte ratio; NLR – neutrophil-to-lymphocyte ratio; PIR – Poverty Income Ratio; TyG – triglyceride-glucose index; TyG-BMI – triglyceride-glucose–body mass index; TyG-WC – triglyceride-glucose–waist circumference; TyG-WHtR – triglyceride-glucose–waist-to-height ratio.

Table S4. Characteristics of NHANES participants between 2007-2018 with imputation data.

Characteristic	Overall (N = 5,067)	Non-gout (N = 4,506, 89.5%)	Gout (N = 561, 10.5%)	p-value
Age	59.65 (13.69)	59.00 (13.83)	65.10 (10.97)	<0.001
Older (≥ 60 years)	3,142.0 (53.0%)	2,706.0 (50.9%)	436.0 (70.7%)	<0.001
Gender (male)	2,554.0 (49.7%)	2,177.0 (47.9%)	377.0 (64.8%)	<0.001
Race				<0.001
Mexican American	859.0 (9.7%)	812.0 (10.3%)	47.0 (4.2%)	
Non-Hispanic Black	1,295.0 (14.2%)	1,120.0 (14.1%)	175.0 (15.8%)	
Non-Hispanic White	1,915.0 (62.3%)	1,659.0 (61.3%)	256.0 (70.6%)	
Other Hispanic	541.0 (5.9%)	501.0 (6.1%)	40.0 (4.0%)	
Other/multiracial	457.0 (7.9%)	414.0 (8.1%)	43.0 (5.5%)	
Education duration (> 12 years)	3,453.0 (77.7%)	3,041.0 (77.0%)	412.0 (83.7%)	0.002
Marital status (married)	2,782.0 (58.5%)	2,445.0 (57.8%)	337.0 (64.4%)	0.038
PIR				0.235
< 1.3	1,750.0 (25.4%)	1,564.0 (25.4%)	186.0 (25.2%)	
1.3-3.5	1,245.0 (35.3%)	1,096.0 (34.8%)	149.0 (40.0%)	
≥ 3.5	2,072.0 (39.3%)	1,846.0 (39.8%)	226.0 (34.8%)	

Characteristic	Overall (N = 5,067)	Non-gout (N = 4,506, 89.5%)	Gout (N = 561, 10.5%)	p-value
Obesity	3,009.0 (64.3%)	2,636.0 (63.4%)	373.0 (72.3%)	0.008
Current drink	2,591.0 (53.2%)	2,282.0 (52.8%)	309.0 (56.0%)	0.325
Current smoke	801.0 (15.4%)	738.0 (16.2%)	63.0 (8.2%)	<0.001
Physical Activity (>600METs/week)	2,188.0 (47.6%)	1,986.0 (48.9%)	202.0 (36.9%)	0.002
Hypertension	3,385.0 (65.8%)	2,917.0 (63.7%)	468.0 (83.1%)	<0.001
Dyslipidemia	4,151.0 (83.1%)	3,678.0 (82.7%)	473.0 (86.7%)	0.120
CVD	1,294.0 (24.7%)	1,048.0 (23.0%)	246.0 (39.5%)	<0.001
CKD	963.0 (15.9%)	741.0 (13.8%)	222.0 (33.8%)	<0.001
Insulin	21.43 (27.94)	21.35 (28.40)	22.09 (23.54)	0.196
TyG	9.15 (0.72)	9.14 (0.72)	9.29 (0.75)	0.150
HOMA-IR	8.39 (13.22)	8.24 (13.09)	9.66 (14.27)	0.191
TyG-BMI	300.27 (74.15)	297.26 (71.55)	326.11 (89.58)	0.016
TyG-WC	1,012.23 (180.75)	1,003.41 (175.34)	1,091.06 (207.64)	0.001
TyG-WHtR	6.06 (1.06)	6.02 (1.04)	6.41 (1.19)	0.017
HEI-2020	51.88 (11.55)	51.93 (11.54)	51.42 (11.63)	0.537

Characteristic	Overall (N = 5,067)	Non-gout (N = 4,506, 89.5%)	Gout (N = 561, 10.5%)	p-value
aMED	3.41 (1.27)	3.42 (1.28)	3.35 (1.24)	0.416
DASH	22.57 (4.77)	22.59 (4.77)	22.36 (4.81)	0.521
AHEI	39.17 (10.70)	39.15 (10.72)	39.35 (10.57)	0.928
DII	2.90 (2.08)	2.91 (2.08)	2.80 (2.07)	0.276
NLR	2.47 (1.40)	2.43 (1.34)	2.85 (1.80)	<0.001
MLR	0.30 (0.14)	0.30 (0.14)	0.34 (0.15)	<0.001
CRP	6.46 (10.93)	6.24 (10.34)	8.37 (14.91)	0.242

Abbreviations: AHEI – Alternative Healthy Eating Index; aMED – alternate Mediterranean diet; CKD – chronic kidney disease; CRP – C-reactive protein; CVD – cardiovascular disease; DASH – Dietary Approaches to Stop Hypertension; DII – Dietary Inflammatory Index; HEI-2020 – Healthy Eating Index-2020; HOMA-IR – homeostatic model assessment for insulin resistance; MLR – monocyte-to-lymphocyte ratio; NLR – neutrophil-to-lymphocyte ratio; PIR – Poverty Income Ratio; TyG – triglyceride-glucose index; TyG-BMI – triglyceride-glucose–body mass index; TyG-WC – triglyceride-glucose–waist circumference; TyG-WHtR – triglyceride-glucose–waist-to-height ratio.

Table S5. The odds ratios between dietary pattern and gout in diabetes patients, NHANES 2007-2018 with imputation data

Dietary patterns	HEI-2020		AHEI		DASH		aMED		DII	
	OR (95% CI)	p-value	OR (95% CI)	p-value	OR (95% CI)	p-value	OR (95% CI)	p-value	OR (95% CI)	p-value
**Per-SD increment**	11.73		10.56		4.63		1.28		2.10	
Crude model	0.96 (0.86, 1.07)	0.458	1.02 (0.91, 1.14)	0.742	0.95 (0.84, 1.06)	0.412	0.95 (0.85, 1.06)	0.384	0.94 (0.82, 1.08)	0.401
Model 1	0.84 (0.75, 0.94)	0.012	0.89 (0.78, 1.01)	0.086	0.83 (0.73, 0.95)	0.015	0.84 (0.75, 0.94)	0.011	1.05 (0.90, 1.22)	0.534
Model 2	0.83 (0.74, 0.93)	0.009	0.89 (0.78, 1.01)	0.092	0.82 (0.72, 0.94)	0.012	0.84 (0.75, 0.94)	0.010	1.05 (0.90, 1.22)	0.518
Model 3	0.81 (0.72, 0.91)	0.008	0.94 (0.81, 1.09)	0.426	0.82 (0.72, 0.93)	0.018	0.85 (0.76, 0.95)	0.024	1.02 (0.84, 1.24)	0.857

Abbreviations: AHEI – Alternative Healthy Eating Index; aMED – alternate Mediterranean diet; CI – confidence interval; CVD – cardiovascular disease; DASH – Dietary Approaches to Stop Hypertension; DII – Dietary Inflammatory Index; HEI-2020 – Healthy Eating Index-2020; OR – odds ratio; SGLT2 – sodium-glucose cotransporter 2.
Model 1: Adjusted for age (years), gender, race/ethnicity, education duration, and marital status.
Model 2: Adjusted for Model 1 plus alcohol consumption, smoking status, leisure-time physical activity, and total energy intake (kcal/day).
Model 3: Adjusted for Model 2 plus hypertension, CVD, dyslipidemia, obesity, and medication use (including urate-lowering drugs, diuretics, SGLT2 inhibitors, and other anti-diabetic medications).

Table S6. Mediation analysis of dietary patterns on gout*

Exposure	Mediator	a-path	b-path	Total effect	Direct effect	Indirect effect	Mediation proportion	P-value
DASH	BMI	-0.78 (-1.11, -0.46)	1.03 (1.00, 1.07)	0.85 (0.69, 1.05)	0.87 (0.70, 1.07)	-0.03 (-0.06, 0.00)	15.3% (-2.7%, 33.4%)	0.096

	SUA	-0.02 (-0.04, 0.00)	3.09 (2.06, 4.64)	0.86 (0.70, 1.07)	0.89 (0.72, 1.10)	-0.02 (-0.04, 0.00)	13.4% (-2.0%, 28.7%)	0.087
	NLR	-0.12 (-0.18, -0.07)	1.10 (1.02, 1.19)	0.86 (0.70, 1.06)	0.87 (0.71, 1.08)	-0.01 (-0.02, -0.00)	8.0% (0.7%, 15.4%)	0.032
	eGFR	1.80 (0.99, 2.61)	0.99 (0.98, 1.00)	0.86 (0.70, 1.07)	0.88 (0.70, 1.10)	-0.02 (-0.03, 0.00)	10.8% (-0.6%, 22.3%)	0.064
	TyG	-0.03 (-0.07, 0.01)	1.59 (1.19, 2.14)	0.90 (0.74, 1.09)	0.91 (0.75, 1.11)	-0.015 (-0.034, 0.005)	14.0% (-4.4%, 32.4%)	0.137
	TyG-BMI	-6.82 (-10.79, -2.86)	1.01 (1.00, 1.01)	0.88 (0.72, 1.09)	0.93 (0.74, 1.16)	-0.036 (-0.073, 0.001)	28.9% (-0.7%, 58.5%)	0.055
	TyG-WC	-17.56 (-27.57, -7.55)	1.00 (1.00, 1.00)	0.90 (0.72, 1.11)	0.94 (0.75, 1.18)	-0.042 (-0.086, 0.002)	39.3% (-1.4%, 80.1%)	0.059
	TyG-WHtR	-0.12 (-0.18, -0.06)	1.49 (1.08, 2.06)	0.89 (0.72, 1.11)	0.94 (0.75, 1.18)	-0.046 (-0.090, -0.002)	40.2% (2.1%, 78.3%)	0.039
HEI-2020	BMI	-0.74 (-1.04, -0.44)	1.03 (0.99, 1.07)	0.83 (0.71, 0.97)	0.85 (0.72, 0.99)	-0.02 (-0.05, 0.00)	12.5% (-2.6%, 27.5%)	0.105
	SUA	-0.00 (-0.02, 0.02)	3.12 (2.08, 4.69)	0.83 (0.70, 0.97)	0.83 (0.71, 0.98)	-0.00 (-0.03, 0.02)	1.9% (-10.3%, 14.1%)	0.762

	NLR	-0.12 (-0.18, -0.07)	1.10 (1.02, 1.19)	0.82 (0.70, 0.96)	0.83 (0.71, 0.97)	-0.01 (-0.02, -0.00)	6.0% (0.5%, 11.5%)	0.033
	TyG	-0.03 (-0.06, 0.00)	1.59 (1.18, 2.14)	0.83 (0.70, 0.99)	0.84 (0.70, 1.00)	-0.014 (-0.032, 0.004)	7.7% (-2.1%, 17.4%)	0.123
	TyG-BMI	-5.89 (-10.02, -1.75)	1.01 (1.00, 1.01)	0.83 (0.70, 1.00)	0.87 (0.73, 1.04)	-0.031 (-0.065, 0.003)	16.9% (-1.7%, 35.4%)	0.074
	TyG-WC	-16.79 (-26.03, -7.55)	1.00 (1.00, 1.00)	0.84 (0.69, 1.02)	0.88 (0.72, 1.07)	-0.040 (-0.081, 0.001)	23.2% (-0.9%, 47.2%)	0.059
	TyG-WHtR	-0.10 (-0.15, -0.04)	1.49 (1.08, 2.05)	0.84 (0.69, 1.03)	0.88 (0.72, 1.07)	-0.039 (-0.077, -0.001)	22.8% (0.7%, 44.9%)	0.044
aMED	eGFR	1.98 (1.04, 2.93)	0.99 (0.98, 1.00)	0.83 (0.70, 0.97)	0.85 (0.71, 1.01)	-0.02 (-0.04, 0.00)	8.8% (-0.9%, 18.4%)	0.075
	BMI	-0.70 (-1.00, -0.40)	1.03 (1.00, 1.07)	0.86 (0.74, 0.99)	0.88 (0.75, 1.02)	-0.02 (-0.05, 0.00)	14.9% (-2.8%, 32.6%)	0.099
	SUA	-0.02 (-0.04, -0.00)	3.09 (2.07, 4.63)	0.87 (0.74, 1.01)	0.89 (0.76, 1.05)	-0.02 (-0.05, -0.00)	16.3% (0.5%, 32.0%)	0.043
	NLR	-0.11 (-0.17, -0.05)	1.10 (1.02, 1.19)	0.86 (0.74, 1.00)	0.87 (0.75, 1.01)	-0.01 (-0.02, -0.00)	7.1% (0.5%, 13.7%)	0.036

	TyG	-0.01 (-0.05, 0.02)	1.60 (1.19, 2.15)	0.82 (0.70, 0.97)	0.82 (0.70, 0.98)	-0.007 (-0.025, 0.011)	3.5% (-5.9%, 12.9%)	0.468
	TyG-BMI	-3.86 (-7.95, 0.24)	1.01 (1.00, 1.01)	0.82 (0.69, 0.98)	0.86 (0.72, 1.02)	-0.020 (-0.047, 0.007)	10.4% (-3.6%, 24.5%)	0.145
	TyG-WC	-12.94 (-22.74, -3.14)	1.00 (1.00, 1.00)	0.85 (0.71, 1.01)	0.88 (0.74, 1.06)	-0.031 (-0.066, 0.005)	18.4% (-2.8%, 39.7%)	0.089
	TyG-WHtR	-0.08 (-0.14, -0.02)	1.48 (1.08, 2.04)	0.84 (0.70, 1.00)	0.87 (0.73, 1.05)	-0.031 (-0.064, 0.002)	17.7% (-1.2%, 36.6%)	0.066

*All data were derived from NHANES and analysed using survey-weighted generalized linear models to account for the complex sampling design. The a-path represents the association between dietary scores and mediators and is reported as β coefficients (95% CI) from linear regression; the b-path represents the association between mediators and prevalent gout and is reported as ORs (95% CIs) from logistic regression. Total, direct, and indirect effects are presented on the log-odds scale. Mediation proportions were calculated as (indirect effect / total effect) × 100%, with 95% CIs derived using the delta method. All models were adjusted for age, sex, race/ethnicity, education level, marital status, smoking status, alcohol consumption, physical activity, total energy intake, hypertension, cardiovascular disease, dyslipidemia, and medication use. P values refer to the mediation effect (indirect effect) tested using the Sobel method, with P < 0.05 considered statistically significant.

Abbreviations: aMED – alternate Mediterranean diet; BMI – body mass index; CI – confidence interval; DASH – Dietary Approaches to Stop Hypertension; eGFR – estimated glomerular filtration rate; HEI-2020 – Healthy Eating Index-2020; NLR – neutrophil-to-lymphocyte ratio; OR – odds ratio; SUA – serum uric acid; TyG – triglyceride-glucose index; TyG-BMI – triglyceride-glucose – body mass index; TyG-WC – triglyceride-glucose – waist circumference; TyG-WHtR – triglyceride-glucose – waist-to-height ratio.

Table S7. Mediation analysis of dietary patterns on gout with imputation data.*

Exposure	Mediator	a-path	b-path	Total effect	Direct effect	Indirect effect	Mediation proportion	P value
DASH	BMI	-0.79 (-1.08, -0.50)	1.03 (1.01, 1.06)	0.85 (0.71, 1.02)	0.87 (0.72, 1.05)	-0.03 (-0.05, -0.01)	16.1% (2.5%, 29.8%)	0.042
	SUA	-0.02 (-0.04, -0.01)	3.09 (2.15, 4.45)	0.86 (0.72, 1.04)	0.89 (0.75, 1.08)	-0.02 (-0.04, -0.00)	14.2% (1.5%, 26.9%)	0.038
	NLR	-0.12 (-0.17, -0.08)	1.10 (1.03, 1.18)	0.86 (0.72, 1.03)	0.87 (0.73, 1.06)	-0.01 (-0.02, -0.00)	8.5% (1.2%, 15.8%)	0.025
	eGFR	1.82 (1.10, 2.54)	0.99 (0.98, 1.00)	0.86 (0.72, 1.04)	0.88 (0.73, 1.07)	-0.02 (-0.03, -0.00)	11.5% (0.5%, 22.5%)	0.048
	TyG	-0.03 (-0.07, 0.01)	1.59 (1.19, 2.14)	0.90 (0.74, 1.09)	0.91 (0.75, 1.11)	-0.015 (-0.034, 0.005)	14.0% (-4.4%, 32.4%)	0.137
	TyG-BMI	-6.82 (-10.79, -2.86)	1.01 (1.00, 1.01)	0.88 (0.72, 1.09)	0.93 (0.74, 1.16)	-0.036 (-0.073, 0.001)	28.9% (-0.7%, 58.5%)	0.055
	TyG-WC	-17.56 (-27.57, -7.55)	1.00 (1.00, 1.00)	0.90 (0.72, 1.11)	0.94 (0.75, 1.18)	-0.042 (-0.086, 0.002)	39.3% (-1.4%, 80.1%)	0.059
	TyG-WHtR	-0.12 (-0.18, -0.06)	1.49 (1.08, 2.06)	0.89 (0.72, 1.11)	0.94 (0.75, 1.18)	-0.046 (-0.090, -0.002)	40.2% (2.1%, 78.3%)	0.039
HEI-2020	BMI	-0.75 (-1.00, -0.50)	1.03 (1.00, 1.06)	0.83 (0.73, 0.95)	0.85 (0.74, 0.97)	-0.02 (-0.04, -0.01)	13.8% (1.5%, 26.1%)	0.044
	SUA	-0.01 (-0.02, 0.01)	3.12 (2.15, 4.50)	0.83 (0.72, 0.95)	0.83 (0.72, 0.96)	-0.00 (-0.02, 0.01)	2.5% (-8.5%, 13.5%)	0.654
	NLR	-0.12 (-0.17, -0.07)	1.10 (1.03, 1.18)	0.82 (0.72, 0.94)	0.83 (0.73, 0.95)	-0.01 (-0.02, -0.00)	6.5% (0.8%, 12.2%)	0.028
	TyG	-0.03 (-0.06, 0.00)	1.59 (1.18, 2.14)	0.83 (0.70, 0.99)	0.84 (0.70, 1.00)	-0.014 (-0.032, 0.004)	7.7% (-2.1%, 17.4%)	0.123
	TyG-BMI	-5.89 (-10.02, -1.75)	1.01 (1.00, 1.01)	0.83 (0.70, 1.00)	0.87 (0.73, 1.04)	-0.031 (-0.065, 0.003)	16.9% (-1.7%, 35.4%)	0.074
	TyG-WC	-16.79 (-26.03, -7.55)	1.00 (1.00, 1.00)	0.84 (0.69, 1.02)	0.88 (0.72, 1.07)	-0.040 (-0.081, 0.001)	23.2% (-0.9%, 47.2%)	0.059
	TyG-WHtR	-0.10 (-0.15, -0.04)	1.49 (1.08, 2.05)	0.84 (0.69, 1.03)	0.88 (0.72, 1.07)	-0.039 (-0.077, -0.001)	22.8% (0.7%, 44.9%)	0.044
aMED	eGFR	2.01 (1.15, 2.87)	0.99 (0.98, 1.00)	0.83 (0.72, 0.95)	0.85 (0.73, 0.99)	-0.02 (-0.04, -0.00)	9.5% (0.5%, 18.5%)	0.046
	BMI	-0.71 (-0.95, -0.47)	1.03 (1.01, 1.06)	0.86 (0.76, 0.97)	0.88 (0.77, 1.00)	-0.02 (-0.04, -0.01)	15.5% (1.5%, 29.5%)	0.041
	SUA	-0.02 (-0.04, -0.01)	3.09 (2.15, 4.45)	0.87 (0.76, 0.99)	0.89 (0.78, 1.02)	-0.02 (-0.04, -0.00)	17.2% (1.5%, 32.9%)	0.032
	NLR	-0.11 (-0.16, -0.06)	1.10 (1.03, 1.18)	0.86 (0.76, 0.98)	0.87 (0.77, 0.99)	-0.01 (-0.02, -0.00)	7.8% (0.8%, 14.8%)	0.029
	TyG	-0.01 (-0.05, 0.02)	1.60 (1.19, 2.15)	0.82 (0.70, 0.97)	0.82 (0.70, 0.98)	-0.007 (-0.025, 0.011)	3.5% (-5.9%, 12.9%)	0.468
	TyG-BMI	-3.86 (-7.95, 0.24)	1.01 (1.00, 1.01)	0.82 (0.69, 0.98)	0.86 (0.72, 1.02)	-0.020 (-0.047, 0.007)	10.4% (-3.6%, 24.5%)	0.145
	TyG-WC	-12.94 (-22.74, -3.14)	1.00 (1.00, 1.00)	0.85 (0.71, 1.01)	0.88 (0.74, 1.06)	-0.031 (-0.066, 0.005)	18.4% (-2.8%, 39.7%)	0.089
	TyG-WHtR	-0.08 (-0.14, -0.02)	1.48 (1.08, 2.04)	0.84 (0.70, 1.00)	0.87 (0.73, 1.05)	-0.031 (-0.064, 0.002)	17.7% (-1.2%, 36.6%)	0.066

*All data were derived from the NHANES database and analysed using survey-weighted generalized linear models to account for the complex sampling design. The a-path represents the association between dietary scores and mediators and is reported as β coefficients (95% CI) from linear regression; the b-path represents the association between mediators and prevalent gout and is reported as ORs (95% CIs) from logistic regression. Total, direct, and indirect effects are presented on the log-odds scale. Mediation proportions were calculated as (indirect effect / total effect) × 100%, with 95% CIs derived using the delta method. All models were adjusted for age, sex, race/ethnicity, education level, marital status, smoking status, alcohol consumption, physical activity, total energy intake, hypertension, cardiovascular disease, dyslipidemia, and medication use. P values refer to the mediation effect (indirect effect) tested using the Sobel method, with $P < 0.05$ considered statistically significant.

Abbreviations: aMED – alternate Mediterranean diet; BMI – body mass index; CI – confidence interval; DASH – Dietary Approaches to Stop Hypertension; eGFR – estimated glomerular filtration rate; HEI-2020 – Healthy Eating Index-2020; NLR – neutrophil-to-lymphocyte ratio; OR – odds ratio; SUA – serum uric acid; TyG – triglyceride-glucose index; TyG-BMI – triglyceride-glucose–body mass index; TyG-WC – triglyceride-glucose–waist circumference; TyG-WHtR – triglyceride-glucose–waist-to-height ratio.

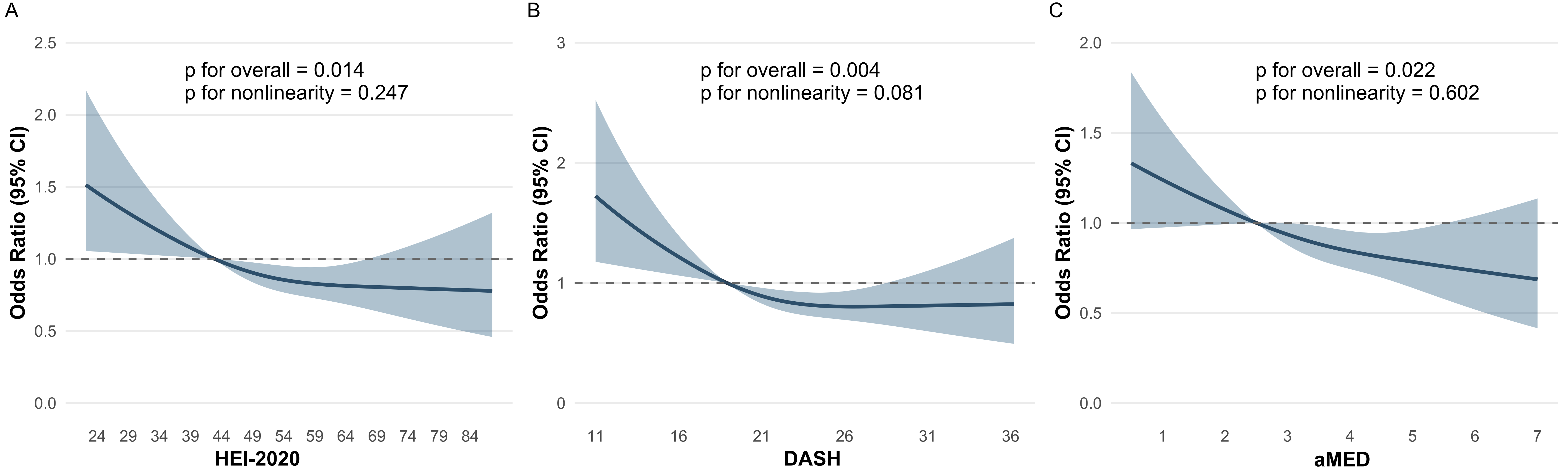

A
p for overall = 0.014
p for nonlinearity = 0.247
Odds Ratio (95% CI)
HEI-2020
B
p for overall = 0.004
p for nonlinearity = 0.081
Odds Ratio (95% CI)
DASH
C
p for overall = 0.022
p for nonlinearity = 0.602
Odds Ratio (95% CI)
aMED
